# Nutrition Knowledge Varies by Food Group and Nutrient Among Adults

**DOI:** 10.3390/foods14040606

**Published:** 2025-02-12

**Authors:** Abigail A. Glick, Donna M. Winham, Michelle M. Heer, Andrea M. Hutchins, Mack C. Shelley

**Affiliations:** 1 Department of Food Science & Human Nutrition, Iowa State University, Ames, IA 50011, USA; aglick03@gmail.com (A.A.G.); mmheer@gmail.com (M.M.H.); 2 Department of Human Physiology & Nutrition, University of Colorado Colorado Springs, Colorado Springs, CO 80918, USA; ahutchin@uccs.edu; 3 Departments of Political Science and Statistics, Iowa State University, Ames, IA 50011, USA; mshelley@iastate.edu

**Keywords:** consumer knowledge, Dietary Guidelines for Americans, chronic disease, carbohydrates, pulses, health benefits of food, whole grains, potassium, folate, dairy

## Abstract

The United States Dietary Guidelines for Americans (DGA) have provided recommendations for healthy eating patterns to meet nutrient needs and reduce chronic disease risk for decades. However, few Americans fully meet these guidelines, especially regarding five shortfall nutrients, and the vegetable, fruit, pulse, whole grain, and dairy food groups. Nutrition knowledge is a modifiable factor associated with improved dietary behavior, yet it is unclear whether individuals with nutrition-related chronic diseases possess greater knowledge. The study objectives were to (1) describe knowledge of 10 nutrient sources across six food groups, and (2) determine predictive factors for nutrient source and food group knowledge. A national sample of 930 adults from an online panel answered demographic, nutrition-disease knowledge, desired benefits from foods, chronic disease status questions, and identified the presence or absence of 10 nutrients in six food groups. Respondents were 77% White, 51% women, and 70% married, with a mean age of 45 years. Nutrition-disease knowledge was higher for those who were older, women, and highly educated. Having a nutrition-related disease, e.g., heart disease, was not predictive of nutrient-disease knowledge. Whole grains had the highest average nutrient knowledge score (6.26 ± 2.5; range 0–10), while vegetables had the lowest (4.89 ± 2.3). Fat food sources were the best known (3.98 ± 1.5; range 0–6), and folate was the least recognized (2.16 ± 1.4). General linear models of survey variables for the six food groups explained 10.2% to 19.4% of nutrient knowledge variation and described 4.7% to 27.1% of differences in food-source scores for the 10 nutrients. Nutrient-disease knowledge had the most significant influence on nutrient source scores. Gaps in understanding nutrient sources suggest the public needs more applied education.

## 1. Introduction

For several decades, the United States (US) Dietary Guidelines for Americans (DGA) have provided recommendations for healthy eating patterns across the lifespan to meet nutrient needs and reduce chronic disease risk [1]. Despite this messaging, few Americans meet the recommendations for all food groups or specific nutrients. Diets across all age groups consistently show lower than recommended intakes of dairy, pulses (legumes, beans, and peas), whole grains, fruits, and vegetables [1]. These food groups often contain nutrient-rich items, with multiple micronutrients, fewer added sugars, saturated fat, and sodium [2]. The DGA recommends limiting the latter nutrients because they can contribute to chronic disease risks and obesity [1].

The DGA recommends macronutrients, protein, carbohydrates, and fat for health maintenance and the prevention of chronic disease. Protein is essential for muscle mass, organ integrity, and cellular functions [3]. Adequate protein intakes are of particular concern for people with chronic conditions, including type 2 diabetes and osteoporosis [3,4]. Limits on saturated fat and added sugar intakes are advised to prevent excessive weight gain and to reduce cardiovascular disease (CVD) and the risk of type 2 diabetes [3,5].

Many Americans have difficulty consuming enough dietary fiber, calcium, vitamin D, and potassium [1]. The inadequate intake of these nutrients is concerning because of their essential roles in healthy physiology and chronic disease reduction [1]. Fiber aids gastrointestinal function, blood lipid regulation, glucose metabolism, and weight maintenance. Thus, adequate fiber consumption is recommended for individuals with certain digestive issues, CVD, type 2 diabetes, and for weight management [3,5]. Calcium, vitamin D, and potassium are important nutrients for many functions but are most known for bone development and reducing the risk of osteoporosis [1,4]. Potassium intakes are linked with controlling blood pressure and reducing CVD risk [3,5]. Iron insufficiency is alarming for infants and children due to its importance in cognitive development and adequate blood-carrying capacity. Iron and folate intakes lag among pre-menopausal females [6,7]. Both micronutrients are essential in supporting fetal development. Iron deficiency anemia contributes to fatigue and poor health among women [7].

Other researchers have identified predictive factors that may influence nutrition knowledge. Higher education levels, elevated socioeconomic status, being a woman, older age [8], having successfully modified dietary intakes before, e.g., weight loss [9], children in the household, diagnosed with a nutrition-related chronic disease [10], and culture or ethnicity [11] are related to nutrition knowledge. Individuals who have more resources and time to devote to food acquisition and preparation have more positive nutrition behaviors than those with limited resources [12].

Consumers with higher nutrition knowledge may have greater intakes of nutritious foods [8,13]. Public health messaging that increases nutrition knowledge correlates positively with improved dietary behaviors [14]. However, there are different types of nutrition knowledge that may, or may not, empower consumers to make healthy decisions. Awareness of nutrition knowledge messaging does not always mean consumers know which foods to eat [15]. For example, in a study of young adult African Americans, 77% knew heart disease risk was associated with dietary cholesterol intake, but only 37% knew that animal products contained cholesterol [16]. In an Australian study, 15.9% of participants scored high on nutrition knowledge, but the association with knowledge and intake of iron-rich foods was insignificant [7]. The knowledge that certain foods are high in particular nutrients can be tested. Yet many nutrition ‘knowledge’ questions fail to ask if respondents know the connections between those components and the potential for chronic disease. Consumers may be aware vitamin C is high in citrus fruits, yet knowledge about the function of vitamin C in disease protection is often limited to reducing cold symptoms [17]. Knowledge about the linkages between nutrients and a disease state may be insufficient for consumers to make actionable choices. People may recognize the health messages (e.g., “consume less sodium”, “eat more fiber”, etc.), but not know how to actually implement the changes in their daily diet [18]. A third component of nutrition knowledge is self-assessment of nutrient intake compared to health recommendations. In a national survey, nearly 67% of respondents felt they met dietary fiber recommendations, but only 5% did [19]. Middle-aged American women who self-reported as low carbohydrate dieters were found to consume more carbohydrates and fat than their non-dieter peers [20]. In turn, nutrition knowledge does not always translate into healthy behavior due to resource limitations, family priorities, and other social or environmental restrictions [21].

This study examined consumer awareness of food group sources for 10 nutrients of concern within the DGA. The objectives were to (1) describe knowledge of 10 nutrients within six food groups; and (2) determine which factors (demographics, nutrition-disease knowledge, presence of a nutrition-related disease, intentions to increase or decrease a given nutrient, and self-perceptions of health and diet) were predictive of food group, and individual nutrient knowledge scores. We hypothesized that persons with higher nutrition-disease knowledge, older age, more years of education, and who express interest in limiting or increasing nutrients in their diet will have higher food group and nutrient food source knowledge [18].

## 2. Materials and Methods

### 2.1. Study Design and Sample Recruitment

A sample of 1474 adults aged 18–80 who lived on the US mainland in November 2023 started the online survey. The proprietary survey panel service (Survey Monkey^®^ Audience, San Mateo, CA, USA) solicited respondents to match age, gender, and regional quotas for national representativeness. The company provided a small monetary incentive for participant completion of the surveys. Of the 1474 responses provided, 37 did not meet the selection criteria for location or age and did not advance in the survey. Ninety-five cases failed one or both of two integrity checks. Sixty-eight respondents were removed for ‘speeding’ or submitting the survey in under 30% of the completion time median (11 min × 30% = 3.3 min [22]. Participants with missing or conflicting demographic data with that of the Survey Monkey account holder (*n* = 83), pertinent research questions (*n* = 52), nutrient or food group responses (*n* = 149), or conflicting or nonsensical responses (*n* = 60) were excluded. The final sample for analysis was 930.

### 2.2. Survey Development

The online survey included demographics, health and diet quality, food acquisition and desired diet attributes, nutrition-disease knowledge, and identification of 10 nutrients across six food groups. Data on consumption of nutrient-rich foods and the health belief model predictors were reported elsewhere [10]. Age, gender, race, ethnicity, marital status, household size and composition, and education questions came from validated surveys [23,24,25,26]. Participants ranked their health status and diet quality using a 5-point Likert scale (poor = 1, excellent = 5) [24,25]. One or more nutrition-related chronic disease conditions diagnosed by a medical professional (high blood pressure, high cholesterol, heart disease, type 2 diabetes, gastrointestinal disorder, or ‘other’) were reported [23]. Participants were asked which, if any, health benefits they wanted from foods [27]. The five options with known nutrition involvement were weight loss, blood glucose control, digestive, cardiovascular, and bone health. More than one option could be selected. Participants were asked if they were trying to limit six nutrients (sugar, carbohydrates, sodium, saturated fat, calories, and cholesterol) [23,26]. A second question inquired about nutrients they were trying to eat enough of (protein, fiber, vitamin D, vitamin C, calcium, iron, potassium, and folate) [23]. These are nutrients associated with DGA recommendations to limit or increase, respectively, for better health [1].

For food acquisition, participants identified if they or another person were the main food shopper and meal preparer [26]. Main shopper and main food preparer represent control over food provisioning. Self-reported health and diet quality variables characterize beliefs in personal health knowledge and behavior. Use of vitamins and/or supplements in the past 6 months was indicated (yes/no) [23]. Seven questions from validated instruments were included relevant to disease and health (dietary fat, fiber, calories, salt, folate, fruit, and vegetable consumption) [27,28].

### 2.3. Food Group and Nutrient Knowledge Matrix

Two sets of matrix questions with check boxes to indicate the presence of nutrients found in six food groups were completed. Respondents were asked, “In general, which nutrients—if any—might be found in foods from these categories?” No serving size or portion information was provided. The six food groups of milk and milk products (dairy), meat, pulses, whole grains, fruits, and vegetables were presented in randomized row order for each participant [1,29]. Multiple checkbox responses per food group row were allowed with options for ‘none of these nutrients’ and ‘not sure’ available. The columns for the first matrix set were carbohydrates, protein, fat, iron, and calcium. The second matrix set columns were fiber, vitamin D, folate or folic acid, potassium, and vitamin C. The column order was not randomized.

Overall coding for the presence or absence of a nutrient in a food group was based upon commonly consumed items within that group having at least 10% of the daily value for the nutrient. Coding for the presence of fat was based on “low fat” labeling claims from the US Code of Federal Regulations for food labeling [29]. The presence of carbohydrates was from the diabetes carbohydrate counting system of one “carbohydrate choice” [30]. Coding of all other nutrients was derived from the US code of Federal Regulations criteria for food labeling claims for “good source”, “contains”, or “provides” [29]. Serving size and nutrient content of commonly consumed foods within each food group were analyzed using data from the DGA [1], US Department of Agriculture, FoodData Central interactive website [31], and the National Institutes of Health Dietary Supplement Fact Sheets [32]. Flour fortification with folic acid was considered when assessing the presence of folic acid in the whole grain food group. Three Registered Dietitian Nutritionists reviewed the categorization of nutrients included within the food groups. No changes were recommended.

### 2.4. Data Analysis and Transformations

All statistical analyses and transformations were conducted via IBM SPSS (version 26.0, IBM, Armonk, NY, USA). Binomial items were coded 0/1. Respondents who reported having at least one chronic disease condition or risk factor were coded by the value 1 for the presence of a nutrition-related disease or condition. The correct answers to the seven questions on nutrition-disease knowledge were totaled to create a summary score. The normally distributed nutrition-knowledge score was split at the midpoint value of 3, or the 47th percentile, to create a dichotomous variable used in table display. The correct responses to the nutrient matrix questions were summed as a composite score for the six food groups and 10 nutrients.

A general linear regression model was chosen for analysis because it accommodated binomial and continuous independent variables to predict a continuous outcome, in addition to its flexible modeling of different distributions [33,34]. The initial general linear models (GLMs) included 13 core items (Table 1). They were built as a linear model examining the interaction between terms using a type 3 sum of squares [33]. Decisions to include other variables in the GLMs were based on the DGA and common dietetics-based medical nutrition therapy recommendations [1]. For example, the GLM for the pulse food group included the 13 core variables, three nutrients to increase (fiber, protein, and folate), and two health benefits (CVD and diabetes/blood sugar management) [35]. A similar process was employed for the 10 individual nutrient knowledge scores. Other variables were added, including the appropriate binomial nutrient(s) to limit or to increase and, if applicable, the desired health benefit that related to that nutrient (Table 1) [1]. All variables were examined for collinearity when modeled together, maintaining a Variance Inflation Factor (VIF) of less than 10 [33,34]. All models displayed include only variables that significantly affected the outcome variable. Reported *R*^2^ values are the adjusted value.

## 3. Results

### 3.1. National Sample Characteristics

The 2022 US Census distributions for age categories were approximated among the 930 survey respondents: 18–25 (8.8% vs. 8.7% nationally), 26–34 (15.6% vs. 12.3%), and 55–64 (12.9% vs. 12.9%) [36]. There were proportionally more respondents in the 35–54 category (50.4% vs. 25.7%) and fewer individuals within the 65+ group (12.3% vs. 17.4%) comparison to the US overall. The racial and ethnic distribution for respondents was higher for non-Hispanic Whites (73.1% vs. 58.4% nationally), with fewer Hispanics or Latinos (8.5% vs. 19.5%), Asians (8.0% vs. 6.4%), and African Americans (6.2% vs. 13.7%) than in the general US population [37]. In comparing the regional distribution of responses to the US, more respondents were in the Northeast (26.9% vs. 17.1%) and about the same for the Midwest (21.4% vs. 20.6%). There were fewer responses in the West (19.8% vs. 23.6%) and South (31.9% vs. 38.6%) than regional percentages for the US overall [36].

### 3.2. Nutrition-Disease Knowledge Questions

Each of the seven nutrition-disease knowledge questions and the summary means by the low vs. high score binomial are shown in Table 2 [27,28]. Over 60% of respondents were aware that eating less salt, more fiber, and less red meat protected against heart disease, but 12–27% were not sure of these health messages. About 25% of the respondents erroneously chose cholesterol in the diet rather than saturated fats as a cause of elevated blood cholesterol. Only 44% thought that not eating fruits and vegetables could cause chronic diseases. Recognition of folate or folic acid deficiency as a cause of congenital neural tube defects (NTDs) was known by over 37%, but 35% were unsure of the nutrient deficiency responsible for this condition. Thirty-eight percent of respondents incorrectly thought sugar had more calories than fat (29%), and almost 19% were unsure of which nutrient option had the most calories.

### 3.3. Demographics, Health Conditions, Desired Benefits from Food

Table 3 displays demographic, health, and food acquisition variables and contrasts by the nutrition-disease knowledge binomial score. Older respondents, women, and smaller households were more likely to be in the higher nutrition-disease knowledge category (all *p* < 0.001). Those with lower nutrition-disease knowledge were likelier to have children in the household, self-report excellent health, and self-report excellent diet quality (all *p* < 0.001).

Table 4 displays the frequency distributions for the presence of nutrition-related disease conditions, vitamin use, benefits desired from foods, and nutrients limited or increased by the nutrition-disease knowledge binomial. Respondents who desired digestive health, weight loss or management, or CVD benefits, were trying to limit sugar and saturated fat, and increase protein and fiber were more likely to have higher nutrition-disease knowledge. Respondents with lower nutrition-disease knowledge were likelier to report trying to increase vitamin C.

### 3.4. Predictors of the Nutrition-Disease Knowledge Score

The nutrition-disease knowledge score is a central factor and the main predictor of food group and nutrient knowledge scores. The initial GLM for its prediction had an *R*^2^ value of 0.130 (Table 5). Non-significant variables were removed sequentially in order of the least contribution to the GLM. The most parsimonious model, which retained properties of equal variance and goodness of fit (*R*^2^ = 0.133), had seven items. Age, education, seeking CVD benefits, wanting to increase fiber (all *p* < 0.001), and identifying as a woman (*p* = 0.014) had positive influences on the nutrition-disease knowledge score. Wanting to limit cholesterol and a high self-reported health negatively influenced nutrition-disease knowledge (both *p* = 0.001).

### 3.5. Food Group Nutrition Knowledge and Nutrient Source Awareness

**Figure 1:**
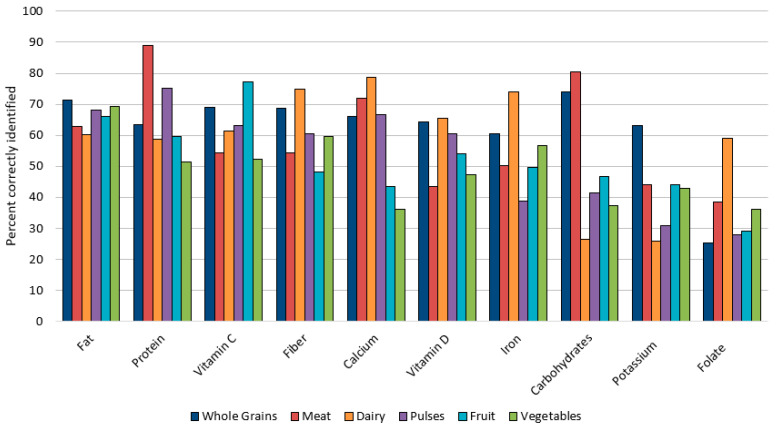
Percentage of adults correctly identifying the presence or absence of 10 nutrients across six food groups.

Figure 1 shows the percentage of respondents correctly identifying the presence or absence of the 10 nutrients across the six food groups. The nutrients are displayed from left to right by the overall percentage identified correctly across the six food groups. The food groups are shown in order of highest average knowledge score for all nutrients.

The mean food group scores were highest for whole grains (6.26 ± 2.5) and lowest for vegetables (4.89 ± 2.3), with the other four food groups ranging between 5.89 and 5.15 (Table 6). The percentage who indicated ‘not sure’ was over 10% for at least five of the 10 nutrients over the six food groups. The percentage who indicated ‘not sure’ was highest for the content of some nutrients in meat (29.8%), fruit (22.7%), and vegetables (19.4%). All food group and nutrient-source mean knowledge scores were significantly different by the nutrient-disease knowledge binomial. Pulses and folate nutrient knowledge were significantly different by presence of a nutrition-related disease (*p* = 0.001; *p* < 0.001).

Summaries of the final GLM for the food group scores are presented in Table 7. Nutrition-disease knowledge positively influenced all food group GLMs (*p* < 0.001), while the effect of other variables differed between food groups. Food group results are described in order of greatest variability explained. The GLM explained 19% (*R*^2^ = 0.194) of the pulse nutrient knowledge scores variation. Other significant predictors were a desire to increase fiber, having a nutrition-related disease, being a woman, acting as the main meal preparer, and trying to increase protein. Vegetable nutrient knowledge scores had an *R*^2^ value of 0.162, and other significant predictors were education level, increasing fiber, and increasing potassium. A similar pattern emerged for the fruit food group with other predictors of higher education, decreasing sugar, increasing fiber, and being the main meal preparer adding to the *R*^2^ value of 0.145. The predictive GLM for dairy (*R*^2^ = 0.140) included seeking bone health benefits, identifying as White, and higher self-reported diet quality, all of which positively influenced dairy knowledge. Whole grains (*R*^2^ = 0.107) included the positive predictor variables of children in the household and a nutrition-related condition, while increased self-reported health negatively predicted whole grains nutrient knowledge. Lastly, married marital status positively predicted meat knowledge (*R*^2^ = 0.077), while older age negatively influenced nutrient-source knowledge for meat.

Table 8 includes the most parsimonious GLMs for predicting knowledge of the sources of macronutrients (carbohydrates, protein, fat, and fiber). Like the food group GLMs, the nutrient-disease knowledge score positively influenced the 10 individual nutrient models (all *p* < 0.001). The fiber GLM had the largest *R*^2^ value (0.271), with predictor variables including a desire for digestive benefits from food, increased age, increasing fiber within the diet, and interest in weight loss management. Other positive predictors for carbohydrate source knowledge (*R*^2^ of 0.230) were education level, an interest in weight loss management, identifying as White, and trying to limit carbohydrate intake. Children in the household were negatively associated with food group knowledge of carbohydrate and fiber sources. In the protein GLM (*R*^2^ = 0.155), the positive predictor variables were trying to increase protein, higher education, being married, and interest in weight loss management. However, negative predictors were older age, children in the household, and interest in limiting cholesterol. The fat GLM had *R*^2^ value of 0.106; besides nutrient-disease knowledge, higher education was a positive predictor. Negative influences on knowledge included having a nutrition-related disease condition, children in the household, and higher self-perceived health status.

Table 9 shows the GLMs for micronutrient knowledge. Nutrient-disease knowledge plays the most significant role for all nutrients (*p* < 0.001). For the vitamin C model, the second predictor was being a woman, and it had the relatively higher *R^2^* value of 0.183 compared to other nutrients. The folate GLM had a *R*^2^ value of 0.121, and its other predictor variables included self-reported diet quality, desire to increase dietary folate, children in the household, a nutrition-related disease condition, and the main meal preparer, all of which positively influenced folate knowledge. The potassium model (*R*^2^ = 0.105) included trying to increase potassium, seeking bone health benefits from the diet, higher education, and presence of children in the household, which all positively influenced knowledge. The iron GLM had *R*^2^ of 0.081, and other predictor variables included seeking energy benefits from food, higher education, being the main meal preparer, and identifying as White. The vitamin D model (*R*^2^ = 0.077) included the two predictor variables of nutrient-disease knowledge and self-reported diet quality. The latter was negatively associated with the vitamin D source knowledge. The calcium model (*R*^2^ = 0.047) positive predictor variables were nutrient-disease knowledge and having children in the household. Trying to increase calcium in the diet and older age were negative influences on calcium knowledge scores.

## 4. Discussion

The study objective was to examine consumer awareness of 10 nutrients in six food groups based on the DGA. We aimed to (1) describe knowledge of nutrient sources and food groups to determine awareness gaps; and (2) determine which factors (demographics, nutrition-disease knowledge, presence of a nutrition-related disease, intentions to increase or decrease a given nutrient, and self-perceptions of health and diet) were predictive of the six food groups and 10 individual nutrient knowledge scores. It was hypothesized that nutrient knowledge would be higher among persons with higher levels of nutrition-disease knowledge and those who are older, have higher education, and express interest in limiting or increasing specific nutrients in their diet.

For the first objective, respondent knowledge was highly variable across food groups and nutrients. Participants had the highest score (63%—6.3 out of 10) for the nutrient content of whole grains. This observation suggests potential success in whole grain nutrition education and marketing. Over two-thirds of participants recognized that whole grains contained carbohydrates and fiber. The USDA School Nutrition Standards require that 80% of grains served in school meals be whole [38]. Parental viewing or engagement with child nutrition programs may be one avenue for their knowledge. A recent systematic review supports this theory, suggesting that institutional lunch interventions with school-aged children positively affect their guardians [39]. However, that does not necessarily mean improved consumption overall, as only 7.9% of adults met the DGA requirement for whole grain intakes in an analysis of National Health and Nutrition Examination Survey (NHANES) data [40].

Nutrient knowledge scores for the meat and dairy food groups were similar to whole grains, at 59%. Over 80% of participants knew meat contained protein and did not have carbohydrates. However, 37% did not realize that meat contained fat. This finding is similar to confusion over animal foods (meat) being a source of cholesterol observed among young adult African Americans [16]. Schupp et al. documented that 84.4% of survey consumers correctly ranked regular ground beef as higher in fat than pork chops or skinless chicken breast [41]. While the current study did not ask about specific types and cuts of meat, over a third of respondents not knowing that meat is a source of fat is concerning. DGA and public health recommendations have emphasized lowering meat intake to reduce chronic disease risk from high fat and greater caloric consumption [1,18,42]. For dairy, about 59–76% knew protein, fat, vitamin D, and calcium were present. This awareness is consistent with messaging about dairy from the DGA and other research [1,4]. However, the low knowledge that dairy products could contain carbohydrates is alarming considering the high prevalence of type 2 diabetes and metabolic syndrome in the US [43,44]. Persons with abnormal glucose metabolism must be aware that fluid milk and many dairy products contain carbohydrate content [45,46].

Food group nutrient knowledge scores were closer to 50% for pulses, fruits, and vegetables. Pulses were recognized for their protein content by 75% of respondents, but fewer recognized their fiber content (61%) compared to whole grains (69%). Most pulses have higher fiber than whole grain products for comparable serving sizes [47]. The vitamin C content of fruits was widely recognized (77%), but knowledge levels of other nutrients were less than 48%. The vegetable knowledge score was the lowest among respondents (49%). Awareness of fiber and iron content in vegetables was only about 50%. The lack of awareness of nutrient density in these food groups suggests campaigns and messaging have been less successful. Education of clients and public health campaigns may focus on particular foods to consume or recommended food patterns rather than specific nutrients in those foods that make them desirable for disease prevention [48]. Results from NHANES data indicate only 10% of US adults meet the recommended intake of 4.5 cups of fruits and vegetables per day [49].

Regarding individual nutrients, knowledge scores were relatively low, with the highest scores of 40% (4/10) for sources of fat and protein. Scores declined from 38% to 21% for the other eight nutrients. The lowest scores were for the identification of carbohydrates, potassium, and folate, with less than half identified correctly. While research is limited on consumer knowledge of carbohydrate sources, it is evident there may be bias against carbohydrates due to fad diets, thus leading to misinformation on the function of this macronutrient [20,50]. Our results suggest a specific misunderstanding of carbohydrate content of dairy, fruit, and vegetables. While we did not compare knowledge levels adjusted by the presence of type 2 diabetes alone, previous studies have suggested individuals with this condition had lower nutrition knowledge [3,51,52]. Siopsis et al. report that while nutrition awareness among persons with type 2 diabetes did not correspond with diet quality, there was a moderate correlation with their self-efficacy score [53]. That study suggests dietary knowledge may enhance the self-efficacy of persons with type 2 diabetes when choosing beneficial foods to consume [53].

Folate, a nutrient of public health concern for women of reproductive age because of its link to fetal neural tube defects, was another nutrient with low food-source knowledge scores. Public health recommendations are “that all persons capable of becoming pregnant get 400 micrograms of folic acid daily” [54]. Other research has shown messaging has successfully increased folic acid supplementation during child-bearing years [54,55]. However, our results indicate individuals lack sufficient knowledge of dietary sources of folate. One recent study examined the relationship between folate and folic acid intake from the diet with overall nutrition knowledge and found that individuals with moderate to high nutrition knowledge were five times more likely to have higher folate consumption [56]. Education programs, especially those focused on at-risk groups such as pregnant women, can also increase folate consumption through food or supplements. However, even after exposure to education campaigns, increased consumption of folate-containing foods or supplements can be variable. Not tailoring the education program to the specific population may account for the variability in adherence to the recommendations [57]. Thus, nutrition knowledge and education on risk factors may increase folate consumption.

Participants had low knowledge of potassium food sources, a shortfall nutrient for many Americans. Potassium is essential for blood pressure regulation, thereby lowering the risk of heart attack and stroke, and critical for bone metabolism, thus reducing the risk of osteoporosis, among other benefits [1]. A previous study of potassium consumption in the US found that intake was lower than recommended amounts [58]. However, prior to 2018, potassium was not required to be present on nutrition facts labels. An analysis of over 6000 labels in 2013 found fewer than 10% provided the potassium content [59]. Since 2018, potassium is required to be on the nutrition facts label, yet public knowledge may still lag due to its previous absence. The emphasis on overall diet patterns in educational campaigns (e.g., Dietary Approaches to Stop Hypertension) may also be responsible for the lack of knowledge of the nutrients in the recommended foods. While campaigns tend to focus on foods to consume more or less of (e.g., eat more fruits and vegetables), they can minimize the reason behind the recommendation (e.g., fruits and vegetables are high in potassium and low in sodium). Therefore, no enhancement of knowledge of food sources of nutrients occurs, even though intake may increase [48].

For our second objective, our study results support both hypotheses that individuals with higher nutrient-disease knowledge and more education had higher food group nutrient and nutrient-source knowledge across all categories. However, when comparing the explained variance of the GLMs across food groups and nutrients, some models, such as those for meat, calcium, vitamin D, and iron explained little of the variation. Since the predictive variables included were theoretically influential in other studies, the model’s weakness may represent a lack of knowledge of these food groups and nutrients. The desire to increase or limit nutrients positively influenced knowledge in most cases. However, there were unexpected conflicts, with reported interest in limiting cholesterol and increasing calcium not being predictive in the respective GLMs for these nutrients. Limited differences in demographic variables such as gender and race were seen when predicting nutrient and food group knowledge, but these variables were antecedents to the nutrition-disease knowledge variable. Women did have higher knowledge of pulse nutrients and vitamin C sources. Identifying as White compared to non-White was predictive of higher dairy, carbohydrate, and iron knowledge. Older age was positively associated with knowledge of certain nutrients such as fiber but primarily was predictive of lower knowledge scores for meat, protein, and calcium. Individuals with a nutrition-related disease had higher knowledge scores for pulses, whole grain, and folate, and lower scores for fat knowledge. Prevention or management of about half of the nutrition-related diseases reported (CVD, type 2 diabetes) is based on monitoring fat intakes. Having a nutrition-related disease lacks significance in the majority of predictive knowledge models. This observation suggests participants may be unable to connect most nutrients and food groups as causes of their chronic diseases [18].

While predictors such as being a woman, higher education, identifying as White, and positive diet modifications agree with previous research, the lack of associations with increasing age, wanting to increase calcium, and wanting to limit cholesterol disagree with earlier studies on nutrition knowledge [8,9,11]. The desire to decrease cholesterol may indicate these individuals are basing dietary recommendations on outdated information. Recommendations that are more recent suggest CVD risk is not significantly correlated with dietary cholesterol, but rather with saturated fat content of the diet [42]. Thus, the limitations on dietary cholesterol have been removed from the DGA [60]. In the current study, age was positively associated with fiber knowledge, which agrees with previous findings [61], suggesting messaging regarding the importance of fiber has been successful. Men and people who identified as non-White had lower nutrient food-source knowledge than shown in other studies with pulses [62]. If greater age, being male, and of non-White ethnic background are negatively associated with knowledge of specific nutrients and food groups, further research is needed. Characterizing perceptions about these items is critical to guide tailored educational interventions to inform people [63]. Some considerations would be cultural, economic, and socially appropriate suggestions or information. If possible, for one-on-one counseling, plans should incorporate intrapersonal-related factors such as taste preferences [63,64].

The GLMs also included variables of interest for respective nutrients. While variables for beneficial diet modifications yielded an overall positive trend for improved nutrient knowledge, a few did not yield significant results or had a negative impact on food source knowledge. Contrary to expectations, respondents who said they were interested in modifying intakes of a particular nutrient were not always knowledgeable of the sources of that nutrient [18,20]. For example, reporting that one was limiting saturated fat and cholesterol intake in the diet was not significantly associated with fat source knowledge. Paradoxically, the desire to increase dietary calcium negatively predicted calcium food-source knowledge. While these results conflicted across various nutrients, it is known that intentions to change diet are not always predictive of behavior or knowledge. One study found that university students who had intentions of eating healthier did not have adequate knowledge of recommendations for their sex and age cohorts. Furthermore, they lacked coping self-efficacy to make these changes regardless of their intentions [65]. Ultimately, while intentions to improve certain nutrients proved to be a positive indicator of nutrition knowledge, our findings support previous work that suggests intentions alone may not be enough without self-efficacy and social support [9,10,15,20].

## 5. Limitations

While there are many strengths to this study, there are also several limitations. Self-reported data were collected from national survey online panelists. Despite the use of two data reliability checks, and exclusion of other cases with incomplete or nonsensical responses, bias may exist in these data due to social desirability in responses, or self-selection because of interest in the topic. Survey instruments assume participants understand the questions in the survey instrument and answer accurately [64]. Further research should include focus groups or observational studies to reduce response bias for which participants are randomly sampled. Data on several topics relevant to nutrition knowledge were not collected. These include participant’s source of nutrition knowledge, health literacy level, income, dietary intakes, use of food nutrition labels, and knowledge of serving recommendations [8,9,12]. For those with a nutrition-related disease, information on nutrition education and time since diagnosis was not asked. Thus, it is not clear if having a diagnosis of a nutrition-related disease, and subsequent treatment or lifestyle changes to manage the disease result in increased nutrition knowledge.

## 6. Conclusions

This study provides quantitative evidence of the level of knowledge for food groups, macronutrients, and micronutrients, to aid health promotion and disease prevention across the life cycle. Higher nutrition knowledge for whole grains, fats, protein, and vitamin C was observed compared to other items evaluated. Knowledge was much lower for identification of nutrients in fruits and vegetables, and food group sources of carbohydrates, folate, and potassium. Survey participants showed disconnect between their expressed intention to increase or decrease a nutrient and the sources of that nutrient. Persons with a nutrition-related disease did not consistently appear to have greater nutrition-disease knowledge. Nutrition knowledge alone may not improve health behavior, but it is certainly a limiting factor in decision-making. Public health nutrition professionals, including registered dietitians, should be aware of these omissions in understanding [35,66]. Although lack of knowledge appears wide spread across demographics, potential high priority target groups for nutrition education are men, people who identify as non-White, and those with lower education. Health promotion and nutrition education should include clear information on the nutrients as well as the food groups that consumers or clients need to know to reduce cognitive gaps in knowledge and application [18]. Future research can build upon the information presented in this study through improved theoretical models leading to better predictive measuring.

## Figures and Tables

**Table 1:** Variables used in general linear models for food group and nutrient knowledge scores.

**Variables Included in All Food Group and Nutrient General Linear Models**	
Continuous	Age, nutrition-disease knowledge score, years of education, self-reported health status, self-reported dietary quality
Binomial	Gender, children in household, race, nutrition-related disease condition, marital status, vitamin use, main shopper, main food preparer
**Food Group and Nutrient Additional Variables:**	
Pulses	Increasing fiber, folate, protein, seeking diabetes management and cardiovascular disease (CVD) benefits
Whole Grains	Increasing fiber and seeking digestive health benefits
Fruit	Increasing fiber, folate, vitamin C, potassium, decreasing sugar and carbohydrates
Vegetables	Increasing, fiber, folate, vitamin C, potassium, and iron
Dairy	Increasing vitamin D, calcium, and seeking bone health benefits
Meat	Increasing protein and decreasing saturated fat and cholesterol
Carbohydrates	Weight loss management, diabetes management, limiting carbohydrates, and limiting sugar
Fat	Weight loss management, diabetes management, limiting carbohydrates, limiting cholesterol, limiting saturated fat, and increasing protein
Protein	Weight loss management, diabetes management, limiting cholesterol, limiting saturated fat, and limiting calories
Fiber	Weight loss management, diabetes management, increasing fiber, seeking CVD benefits, and digestive benefits
Iron	Increasing iron and seeking energy benefits
Calcium	Increasing calcium and seeking bone health benefits
Vitamin C	Increasing vitamin C
Folate	Increasing folate
Vitamin D	Increasing vitamin D and seeking bone health benefits
Potassium	Increasing potassium and seeking bone health benefits

**Table 2:** Responses to nutrition-disease questions by binomial nutrition-disease knowledge score among US adults aged 18–80 years.

	Total (*n* = 930)	Nutrition-Disease knowledge Score 0–3 (47.5%; 442)	Nutrition-Disease Knowledge Score 4–7 (52.5%; 488)	*p*
	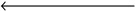 % 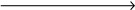			
Eating less salt protects against heart disease **Yes—Correct** No Not sure	79.1 8.2 12.7	64.9 _a_ 13.8 _a_ 21.3 _a_	92.0 _b_ 3.1 _b_ 4.9 _b_	<0.001
Eating more fiber protects against heart disease **Yes—Correct** No Not sure	63.7 8.7 27.6	43.7 _a_ 14.7 _a_ 41.6 _a_	81.8 _b_ 3.3 _b_ 15.0 _b_	<0.001
Eating more meat protects against heart disease **No—Correct** Yes Not sure	60.6 19.2 20.1	33.7 _a_ 32.6 _a_ 33.7 _a_	85.0 _b_ 7.2 _b_ 7.8 _b_	<0.001
Item in foods that raises blood cholesterol level **Saturated fats—Correct** Cholesterol in the diet Polyunsaturated fats Antioxidants Not sure	46.2 24.6 14.8 4.8 9.5	21.3 _a_ 34.8 _a_ 18.8 _a_ 8.4 _a_ 16.7 _a_	68.9 _b_ 15.4 _b_ 11.3 _b_ 1.6 _b_ 2.9 _b_	<0.001
Not eating fruits/vegetables can cause disease **True—Correct** False	43.9 56.1	23.5 _a_ 76.5 _a_	62.3 _b_ 37.7 _b_	<0.001
Nutrient that helps prevent neural tube defects **Folic acid or folate—Correct** Iron Vitamin A Vitamin D Not sure	37.3 9.1 9.1 8.7 35.7	16.3 _a_ 13.3 _a_ 15.1 _a_ 13.8 _a_ 41.5 _a_	56.4 _b_ 5.5 _b_ 3.7 _b_ 4.1 _b_ 30.3 _b_	<0.001
Item that has the most calories **1 g of fat—Correct** 1 g of sugar 1 g of protein 1 g of fiber Not sure	29.0 38.3 8.9 4.8 18.9	14.0 _a_ 39.4 _a_ 14.3 _a_ 8.6 _a_ 23.8 _a_	42.6 _b_ 37.3 _a_ 4.1 _b_ 1.4 _b_ 14.5 _b_	<0.001
Summary score ($\bar{x}$ ± SD)	3.61 ± 1.6	2.19 ± 0.9	4.90 ± 0.9	<0.001

Same subscript letters (a, b) indicate column proportions that are not significantly different. The *p*-value is derived from chi-square analysis.

**Table 3:** Demographic characteristics and perceptions of health and diet status of US adults aged 18–80 by binomial nutrition-disease knowledge score.

	Total (*n* = 930)	Nutrition-Disease Knowledge Score 0–3 (47.5%; 442)	Nutrition-Disease Knowledge Score 4–7 (52.5%; 488)	*p*
	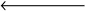 mean ± standard deviation 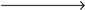			
Age in years	45.1 ± 14.4	41.8 ± 13.6	48.1 ± 14.4	<0.001
Total household size	3.1 ± 1.4	3.4 ± 1.3	2.9 ± 1.3	0.001
	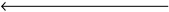 % 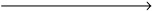			
Gender Men Women	48.7 51.3	54.5 _a_ 45.5 _a_	43.4 _b_ 56.6 _b_	<0.001
Marital Status Single/Divorced/Widowed Married/Living w/partner	28.6 70.4	29.6 70.4	27.7 72.3	^1^ n.s.
Children in household No children One child+ in household	49.2 50.8	41.4 _a_ 58.6 _a_	56.4 _b_ 43.6 _b_	<0.001
Race/Ethnicity Other White	23.2 76.8	25.8 74.2	20.9 79.1	n.s.
Years of Education 9–12th grade and/or GED Some college, no degree Associates degree, Tech school Bachelor’s degree Masters, Doctoral, Professional degree	14.5 13.7 13.8 32.5 25.5	17.0 12.2 12.4 32.8 25.6	12.3 15.2 15.0 32.2 25.4	n.s.
Self-reported health Poor–Fair Good Very good Excellent	17.1 42.7 28.9 11.3	15.6 _a_ 41.2 _a_ 26.9 _a_ 16.3 _a_	18.4 _a_ 44.1 _a_ 30.7 _a_ 6.8 _b_	<0.001
Self-reported diet quality Poor–Fair Good Very good Excellent	21.1 42.2 26.1 10.6	19.5 _a_ 39.1 _a_ 25.3 _a_ 16.1 _a_	22.5 _a_ 44.9 _a_ 26.8 _a_ 5.7 _b_	<0.001
Vitamin and/or supplement use No Yes	23.5 76.5	23.1 76.9	24.0 76.0	n.s.
Main food shopper No Yes	25.7 74.3	26.5 73.5	25.0 75.0	n.s.
Main food preparer No Yes	42.8 57.2	43.4 56.6	42.2 57.8	n.s.

^1^ n.s. = not significant; Same subscript letters (a, b) indicate column proportions that are not significantly different. The *p*-value is derived from chi-square analysis.

**Table 4:** Nutrition-related disease conditions, desired health benefits of foods, and types of nutrient intakes altered by low vs. high nutrition-disease knowledge among adults aged (18–80) (*n* = 930).

	Total	Nutrition-Disease Knowledge 0–3 (47.5%; 442)	Nutrition-Disease Knowledge 4–7 (52.5%; 488)	*p*
	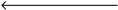 % 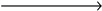			
Binomial nutrition-related disease presence No Yes	44.4 55.6	45.5 54.5	43.4 56.6	^1^ n.s.
Nutrition-related disease conditions High blood pressure High cholesterol Diabetes Gastrointestinal disorder Heart disease	29.8 25.4 17.7 14.7 6.5	28.3 25.3 19.7 10.2 _a_ 7.9	31.1 25.4 16.0 18.9 _b_ 5.1	n.s. n.s. n.s. <0.001 n.s.
Health benefits wanted from foods Weight loss or management Digestive or gut health Heart/cardiovascular health Bone health Diabetes or blood sugar management None of these	49.4 49.9 40.3 35.3 24.7 9.8	45.9 _a_ 41.2 _a_ 33.3 _a_ 36.0 21.9 12.7 _a_	52.5 _b_ 57.8 _b_ 46.7 _b_ 34.6 27.3 7.2 _b_	0.049 <0.001 <0.001 n.s. n.s. 0.006
Trying to limit or eat less nutrients Sugar Carbohydrates Sodium Saturated fat Calories Cholesterol No, not limiting any nutrients	54.9 37.2 36.5 32.2 34.1 31.9 13.8	45.9 _a_ 37.3 33.7 28.3 _a_ 34.6 33.9 14.3	63.1 _b_ 37.1 38.9 35.7 _b_ 33.6 30.1 13.3	<0.001 n.s. n.s. 0.017 n.s. n.s. n.s.
Trying to eat more nutrients Protein Fiber Vitamin D Vitamin C Calcium Iron Potassium Folate No, not increasing any nutrients	45.5 40.8 42.5 39.2 29.7 25.9 19.7 13.0 16.9	38.5 _a_ 31.9 _a_ 43.7 42.8 _a_ 30.5 27.8 19.5 12.0 18.7	51.8 _b_ 48.8 _b_ 41.4 36.1 _b_ 28.9 24.2 19.9 13.9 15.2	<0.001 <0.001 n.s. 0.037 n.s. n.s. n.s. n.s. n.s.

^1^ n.s. = not significant. Same subscript letters (a, b) indicate column proportions that are not significantly different. The *p*-value is derived from chi-square analysis.

**Table 5:** Parameter estimates of a general linear model: measuring the strength of predictor variables on nutrition-disease knowledge (*n* = 930).

Variable	Beta (*p*-Value)	Partial Eta Squared	Observed Power
Age	0.023 (<0.001)	0.045	>0.999
Education	0.162 (<0.001)	0.020	0.991
Seeking cardiovascular disease benefits	0.490 (<0.001)	0.022	0.996
Increasing fiber	0.494 (<0.001)	0.023	0.997
Limiting cholesterol	−0.364 (0.001)	0.011	0.904
Self-reported health status	−0.181 (0.001)	0.011	0.893
Gender (woman)	0.249 (0.014)	0.006	0.689

Model adjusted *R*^2^ = 0.133, *F* value = 21.362, partial eta squared = 0.103, observed power > 0.999, and general linear model *p*-value < 0.001.

**Table 6:** Percentage of adults aged 18–80 who correctly identified or were not sure of the presence or absence of 10 nutrients in six food groups with mean scores for correct placement (*n* = 930).

	Whole Grains	Meat	Dairy	Pulses	Fruit	Vegetables	Nutrient-Source Score
							$\bar{x}$ ± SD
FAT							3.98 ± 1.51
Nutrient source	No	Yes	Yes	No	No	No	
% Correct	71.3	62.7	60.2	68.1	66.0	69.2	
% Not sure	7.7	3.1	2.7	9.1	22.7	19.4	
PROTEIN							3.97 ± 1.42
Nutrient source	No	Yes	Yes	Yes	No	No	
% Correct	63.5	88.9	58.8	75.1	59.6	51.4	
% Not sure	7.7	3.1	2.7	9.1	22.7	19.4	
VITAMIN C							3.78 ± 1.57
Nutrient source	No	No	No	No	Yes	Yes	
% Correct	68.9	54.4	61.5	63.2	77.3	52.4	
% Not sure	16.2	29.8	16.0	20.3	7.2	12.2	
FIBER							3.66 ± 1.70
Nutrient source	Yes	No	No	Yes	Yes	Yes	
% Correct	68.6	54.3	74.8	60.5	48.1	59.6	
% Not sure	16.2	29.8	16.0	20.3	7.2	12.2	
CALCIUM							3.63 ± 1.46
Nutrient source	No	No	Yes	No	No	No	
% Correct	66.2	71.9	78.8	66.6	43.5	36.1	
% Not sure	7.7	3.1	2.7	9.1	22.7	19.4	
VITAMIN D							3.35 ± 1.61
Nutrient source	No	No	Yes	No	No	No	
% Correct	64.4	43.5	65.4	60.6	53.9	47.3	
% Not sure	16.2	29.8	16.0	20.3	7.2	12.2	
IRON							3.30 ± 1.25
Nutrient source	No	Yes	No	Yes	No	Yes	
% Correct	60.6	50.3	74.0	38.8	49.7	56.6	
% Not sure	7.7	3.1	2.7	9.1	22.7	19.4	
CARBOHYDRATES							3.06 ± 1.58
Nutrient source	Yes	No	Yes	Yes	Yes	Yes	
% Correct	73.9	80.3	26.5	41.4	46.7	37.3	
% Not sure	7.7	3.1	2.7	9.1	22.7	19.4	
POTASSIUM							2.50 ± 1.38
Nutrient source	No	No	Yes	Yes	Yes	Yes	
% Correct	63.0	44.0	25.8	30.8	44.1	42.9	
% Not sure	16.2	29.8	16.0	20.3	7.2	12.2	
FOLATE							2.16 ± 1.37
Nutrient source	Yes	No	No	Yes	Yes	Yes	
% Correct	25.2	38.6	58.9	27.8	29.1	36.1	
% Not sure	16.2	29.8	16.0	20.3	7.2	12.2	
FOOD GROUP SCORE $\bar{x}$ ± SD	6.26 ± 2.51	5.89 ± 2.26	5.85 ± 2.15	5.33 ± 2.30	5.15 ± 2.51	4.89 ± 2.30	

**Table 7:** Parameter estimates of a general linear model: measuring the strength of predictor variables on food group nutrient knowledge (*n* = 930).

Nutrient Knowledge Score	Beta (*p* Value)	Partial Eta Squared	Observed Power	Adjusted *R*^2^
PULSES				0.194
Nutrition-Disease Knowledge	0.479 (<0.001)	0.119	>0.999	
Increasing Fiber	0.575 (<0.001)	0.016	0.974	
Nutrition-Related Condition	0.415 (0.003)	0.010	0.852	
Gender	0.388 (0.005)	0.008	0.796	
Main Meal Preparer	0.344 (0.014)	0.007	0.691	
Increasing Protein	0.309 (0.031)	0.005	0.580	
VEGETABLES				
Nutrition-Disease Knowledge	0.452 (<0.001)	0.104	>0.999	0.162
Education	0.235 (<0.001)	0.023	0.997	
Increasing Fiber	0.505 (0.001)	0.013	0.932	
Increasing Potassium	0.447 (0.012)	0.007	0.713	
FRUIT				
Nutrition-Disease Knowledge	0.490 (<0.001)	0.099	>0.999	0.145
Education	0.154 (0.006)	0.008	0.789	
Trying to limit sugar	0.387 (0.015)	0.006	0.686	
Trying to increase fiber	0.388 (0.016)	0.006	0.673	
Main meal preparer	0.325 (0.0360	0.005	0.557	
DAIRY				
Nutrition-Disease Knowledge	0.432 (<0.001)	0.110	>0.999	0.140
Seeking Bone Health Benefits	0.519 (<0.001)	0.015	0.960	
Race/Ethnicity	0.579 (<0.001)	0.015	0.961	
Self-Reported Diet Quality	0.149 (0.034)	0.005	0.563	
WHOLE GRAINS				
Nutrition-Disease Knowledge	0.440 (<0.001)	0.081	>0.999	0.102
Has Children in the Household	0.387 (0.015)	0.006	0.681	
Nutrition-Related Disease	0.373 (0.018)	0.006	0.654	
Self-reported health status	−0.197 (0.023)	0.006	0.624	
MEAT				
Nutrition-Disease Knowledge	0.392 (<0.001)	0.075	>0.999	0.077
Age	−0.014 (0.005)	0.008	0.796	
Marital status	0.392 (0.013)	0.007	0.698	

The *p*-value is derived from comparison of the computed F statistic to the F-distribution under the null hypothesis.

**Table 8:** Parameter estimates of a general linear model: measuring the strength of predictor variables on food group macronutrient knowledge (*n* = 930).

Food Group Macronutrient Knowledge Score	Beta (*p* Value)	Partial Eta Squared	Observed Power	Adjusted *R*^2^
FIBER				0.271
Nutrition-disease knowledge	0.410 (<0.001)	0.158	>0.999	
Digestive benefits	0.359 (<0.001)	0.014	0.948	
Age	0.011 (0.002)	0.011	0.882	
Increasing fiber	0.326 (0.001)	0.011	0.892	
Interested in weight loss	0.271 (0.005)	0.008	0.794	
Children in the household	−0.255 (0.012)	0.007	0.713	
CARBOHYDRATES				0.230
Nutrition-disease knowledge	0.352 (<0.001)	0.140	>0.999	
Education	0.139 (<0.001)	0.018	0.984	
Interested in weight loss	0.385 (<0.001)	0.018	0.983	
Race	0.437 (<0.001)	0.017	0.980	
Children in the household	−0.352 (<0.001)	0.015	0.966	
Eating fewer carbohydrates	0.261 (0.008)	0.008	0.759	
PROTEIN				0.155
Nutrition-disease knowledge	0.274 (<0.001)	0.095	>0.999	
Age	−0.12 (<0.001)	0.014	0.951	
Trying to increase protein	0.342 (<0.001)	0.016	0.970	
Children in the household	−0.318 (0.001)	0.011	0.895	
Education	0.101 (0.002)	0.010	0.876	
Limiting cholesterol	−0.221 (0.020)	0.006	0.641	
Marital status (married)	0.233 (0.026)	0.005	0.603	
Interested in weight loss	0.174 (0.046)	0.004	0.514	
FAT				0.106
Nutrition-disease knowledge	0.263 (<0.001)	0.079	>0.999	
Education	0.088 (0.014)	0.006	0.689	
Nutrition-related disease	−0.226 (0.018)	0.006	0.661	
Children in the household	−0.189 (0.049)	0.004	0.505	
Self-reported health status	−0.104 (0.053)	0.004	0.490	
Interested in weight loss	0.153 (0.105)	0.003	0.368	

The *p*-value is derived from comparison of the computed F statistic to the F-distribution under the null hypothesis.

**Table 9:** Parameter estimates of a general linear model: measuring the strength of predictor variables on food group micronutrient knowledge (*n* = 930).

Food Group Micronutrient Knowledge Score	Beta (*p* Value)	Partial Eta Squared	Observed Power	Adjusted *R*^2^
VITAMIN C				0.183
Nutrition-disease knowledge	0.399 (<0.001)	0.171	>0.999	
Gender	0.249 (<0.001)	0.008	0.754	
FOLATE				0.121
Nutrition-disease knowledge	0.207 (<0.001)	0.063	>0.999	
Self-reported Diet Quality	0.174 (<0.001)	0.015	0.966	
Trying to increase Folate	0.508 (<0.001)	0.017	0.977	
Children in the Household	0.260 (0.001)	0.010	0.847	
Nutrition Related Condition	0.229 (0.002)	0.008	0.759	
Main Meal Preparer	0.200 (0.020)	0.006	0.631	
POTASSIUM				0.105
Nutrition-disease knowledge	0.219 (<0.001)	0.067	>0.999	
Increasing Potassium	0.417 (<0.001)	0.016	0.968	
Seeking Bone Health Benefits	0.267 (0.003)	0.009	0.833	
Education	0.082 (0.010)	0.007	0.734	
Children in the Household	0.189 (0.030)	0.005	0.581	
IRON				0.081
Nutrition-disease knowledge	0.178 (<0.001)	0.053	>0.999	
Seeking Energy Benefits	0.226 (0.005)	0.009	0.804	
Education	0.061 (0.035)	0.005	0.560	
Main Meal Preparer	0.154 (0.054)	0.004	0.486	
Race (White)	0.181 (0.055)	0.004	0.458	
VITAMIN D				0.077
Nutrition-disease knowledge	0.265 (<0.001)	0.072	>0.999	
Self-reported Diet Quality	−0.115 (0.031)	0.005	0.577	
CALCIUM				0.047
Nutrition-disease knowledge	0.123 (<0.001)	0.018	0.985	
Increasing Calcium	−0.384 (<0.001)	0.015	0.959	
Children in the Household	0.343 (<0.001)	0.013	0.931	
Age	−0.008 (0.021)	0.006	0.636	

The *p*-value is derived from comparison of the computed F statistic to the F-distribution under the null hypothesis.

## Data Availability

The data presented in this study are available on request from the corresponding author. The data are not publicly available due to privacy restrictions.
